# Troxerutin Protects Kidney Tissue against BDE-47-Induced Inflammatory Damage through CXCR4-TXNIP/NLRP3 Signaling

**DOI:** 10.1155/2018/9865495

**Published:** 2018-04-19

**Authors:** Qun Shan, Gui-hong Zheng, Xin-rui Han, Xin Wen, Shan Wang, Meng-qiu Li, Juan Zhuang, Zi-Feng Zhang, Bin Hu, Yanqiu Zhang, Yuan-Lin Zheng

**Affiliations:** ^1^School of Environment Science and Spatial Informatics, China University of Mining and Technology, Xuzhou, 221008 Jiangsu, China; ^2^Key Laboratory for Biotechnology on Medicinal Plants of Jiangsu Province, School of Life Science, Jiangsu Normal University, 101 Shanghai Road, Xuzhou, 221116 Jiangsu, China; ^3^College of Health Science, Jiangsu Normal University, 101 Shanghai Road, Xuzhou, 221116 Jiangsu, China

## Abstract

2,2′,4,4′-Tetrabromodiphenyl ether (BDE-47) induces oxidative stress in kidney cells, but the underlying mechanism remains poorly understood. Troxerutin, a natural flavonoid, has potential antioxidant and anti-inflammatory efficacy. In this study, we assessed the effect of troxerutin on kidney damage caused by BDE-47 and investigated the underlying mechanism. The results showed troxerutin reduced reactive oxygen species (ROS) level and urine albumin-to-creatinine ratio (ACR), decreased the activities of inflammatory factors including cyclooxygenase-2 (COX-2), induced nitric oxide synthase (iNOS) and nuclear factor kappa B (NF-*κ*B) in the kidney tissues of BDE-47-treated mice. Furthermore, troxerutin significantly weakened the expression of kidney NLRP3 inflammasome containing NLRP3, ASC, and caspase-1, contributing to the decline of IL-1*β*. Additionally, troxerutin inhibited the increased protein level of stromal-derived factor-1(SDF-1), C-X-C chemokine ligand 12 receptor 4 (CXCR4), and thioredoxin interaction protein (TXNIP) caused by BDE-47. Specifically, the immunoprecipitation assay indicated that there was a direct interaction between CXCR4 and TXNIP. CXCR4 siRNA and TXNIP siRNA also decreased the inflammatory damage, which was similar to the action of troxerutin. Our data demonstrated that troxerutin regulated the inflammatory lesions via CXCR4-TXNIP/NLRP3 inflammasome in the kidney of mice induced by BDE-47.

## 1. Introduction

Oxidative stress and inflammatory response have been confirmed to play vital roles in some clinical diseases such as chronic kidney damage and atherosclerosis [1, 2]. Free radicals are toxic reactive oxygen species (ROS) including superoxide anion, hydroxyl radicals, hydroperoxide, and peroxynitrite. It is well known that there is a dynamic balance between free radical scavenging and generation under normal physiological conditions. However, many diseases frequently increased the production of free radicals, resulting in the cellular oxidative stress and inflammation. Therefore, the intervention of oxidative stress and inflammation may ameliorate kidney damage and contribute to the prevention and treatment of kidney diseases [3].

Polybrominated diphenyl ethers (PBDEs) are synthetic flame retardants generally used in building materials, insulation materials, polyurethane foam, textiles, and plastics. PBDEs are attracting much more attention due to its recalcitrance, effumability, and accumulative toxicity. BDE-47 is one of the most prevalent homologues in the human tissues and environment samples and has a wide distribution and very high content in biological bodies, which mainly accumulates in the liver, kidney, and adipose tissues. Furthermore, BDE-47 induces the toxicity in the tissues of the thyroid gland, liver, kidney, and the cells of spermatocytes and neurons [4–9]. Therefore, it is emerged for the prevention/treatment of toxicity of PBDEs. Recent findings suggest that BDE-47 has the ability to induce oxidative stress and mediate innate immune response and cell apoptosis [10–12]. Interestingly, troxerutin, a natural product flavonoid, may alleviate BDE-47-induced tissue injury [7, 8]. In spite of the widespread occurrence and multiple toxicities of PBDEs, the mechanism underlying in the tissue damage caused by BDE-47 is poorly understood.

Troxerutin, a natural flavonoid, is enriched in fruits, vegetables, tea, coffee, and cereals, possesses high water solubility and is easily absorbed by the gastrointestinal system without toxicity. The accumulating data show that troxerutin owns a wide array of pharmacological activities including antioxidation and anti-inflammation [13, 14] and exhibits the preventive and therapeutic potential on cancer, neuropathic pain, diabetic mellitus, and Alzheimer disease [12, 13, 15]. The previous results in our lab have demonstrated that troxerutin significantly reduced the injury of several tissues including brain, liver, and kidney through decreasing the level of ROS [16–20]. For example, troxerutin improved the memory deficits of mice caused by domoic acid or cholesterol by suppressing inflammatory reactions and oxidative stress [19, 20]. Also, troxerutin markedly inhibited the activation of nucleotide-binding oligomerization domain-like receptor (NLR) protein 3 containing pyrin domain (NLRP3) inflammasome of human umbilical vein endothelial cells (HUVECs) induced by D-galactose [21]. Additionally, troxerutin regulated the lipid metabolism in the liver of high-fat diet-treated mice by blocking oxidative stress-associated NAD^+^ depletion, which especially played the potential protective role in liver damage induced by BDE-47 via targeting the nuclear factor kappa B (NF-*κ*B) [7]. However, it still needs to determine whether troxerutin has a protective function and how it plays a regulatory role in the prevention of mouse kidney inflammatory damage induced by BDE-47.

## 2. Materials and Methods

### 2.1. Animal and Treatment

All experiment procedures were in agreement with Chinese legislation about the use and care of laboratory animals and the animal care committee at the respective university. Male C57BL/6J strain mice aged 6 weeks were obtained from the Branch of National Breeder Center of Rodents (Beijing, China). The mice were provided food and water ad libitum under regular constant conditions. After adaption of one week, the mice were randomly divided into four groups: normal control group (Ctrl), BDE-47-treated group, (BDE-47), troxerutin- and BDE-47-cotreated group (BDE-47/Trox), and troxerutin group (Trox). BDE-47-treated mice were given orally BDE-47 at a dose of 50 mg/kg/day (>99% purity, Chem Service, West Chester, PA, USA; the dose selection of BDE-47 was seen in Supplement Materials (available here)) for 8 weeks (5 days/week), and other groups were given the same dose of corn oil solvent. After four hours of BDE-47 treatment every day, the mice of the BDE-47/troxerutin and troxerutin groups were administrated orally 100 mg/kg/day troxerutin (dissolved in distilled water containing 0.1% Tween 80; >99% purity, Baoji Fangsheng Biotechnology Co. Ltd., Baoji, China; the dose selection is in Supplement Materials), and the mice of the other groups were given an equal solvent. After 8 weeks, the mice were sacrificed to take out the kidneys stored at −80°C for experiments.

C-X-C chemokine ligand 12 (CXCL12) receptor 4 (CXCR4) siRNA or thioredoxin interaction protein (TXNIP) siRNA was used to explore the toxic mechanism of BDE-47 on kidney damage of mice. The mice were randomly divided into four groups: control group, BDE-47-treated group, BDE-47/scramble group, and BDE-47/CXCR4 siRNA group or BDE-47/TXNIP siRNA group. BDE-47-treated mice were given orally BDE-47 at a dose of 50 mg/kg/day for 8 weeks, and other groups were given the same dose of corn oil solvent. After four weeks of BDE-47 supplement, the mice of BDE-47/CXCR4 siRNA were treated 60–100 *μ*l of CXCR4 siRNA 20 *μ*M by tail vein injection for 4 weeks, and the mice of the BDE-47/scramble group were given equivalent scramble mimics (dissolved in sodium phosphate buffer saline (PBS, pH7.2)). As for the latter, the mice of BDE-47/TXNIP siRNA group were simultaneously treated 60–100 *μ*l of TXNIP siRNA 20 *μ*M by tail vein injection every other day for 4 weeks. CXCR4 smart siRNAs (94si, 5′-GAACCGAUCAGUGUGAGUA-3′; 192si, 5′-AACGUCCAUUUCAAUAGG-3′; 420si, 5′-GUGUAAGGCUGUCCAUAUC-3′; and 703si, 5′-GUGUUUCAAUUCCAGCAUA-3′) [22] were pooled to 20 *μ*M, respectively. Two TXNIP siRNAs (targeting 467–487 and 1043–1063 mRNA sites) were pooled to 20 *μ*M, respectively. The scrambled siRNA (20 *μ*M) was used as a control. The siRNA sequences were listed in the following: Tx467siR sense—5′-GCAAACAGACUUUGGACUAUU-3′; Tx467siR antisense—5′-UAGUCCAAAGUCUGUUUGCUU-3′; Tx1043 siR sense—5′-GCCUCAGAGUGCAGAAGAUUU-3′; and Tx1043 siR antisense—5′-AUCUUCUGCACUCUGAGGCUU-3′. The control siRNA sequences are the following: sense 5′-UUCUCCGAACGUGUCACGUUU-3′ and antisense 5′-ACGUGACACGUUCGGAGAAUU-3′.

### 2.2. Urine Collection and Determination of Albumin and Creatinine

The collection and the determination of albumin and creatinine were the same as the previous methods [23]. Urine samples were collected from the mice housed in metabolic cages for 24 h. The excretion of urine protein was evaluated using urine albumin-to-creatinine ratio (ACR) in 24 h urine collections. The content of urine creatinine and albumin was measured using the commercial kits (Jiancheng Institute of Biotechnology, Nanjing, China). The absorbed value was examined by an ultraviolet/visible spectrometer (UV-2501PC, Shimadzu, Japan).

### 2.3. Histological Evaluation

The mice were anesthetized and transcardially perfused with 0.9% sterile saline. After, the kidneys were prefixed with a little of 4% paraformaldehyde (PFA)/PBS (pH 7.4), then removed promptly and postfixed in 4% PFA/PBS (pH 7.4) at 4°C for 4 h, and set successively in 15%, 20%, and 30% sucrose/pH 7.4 PBS solution to make them sink. At last, the kidneys were embedded in optimal cutting temperature compound (Leica, CA, Germany). 12 *μ*m cryosections were collected by using a Leica 3050 (Leica, CA, Germany) for immunofluorescence.

Immunofluorescence was carried out as follows: After 2 h drying at 37°C, the sections were performed to antigen retrieval in boiling 0.1 mol/l sodium citrate buffer including 0.1% Tween-20 for 18 min and incubated in pH 7.2 PBS buffer (including 0.3% Tween-20 and 5% bovine serum albumin (BSA)) at 25°C for 1 h to block nonspecific binding site. Then the sections were incubated overnight with rabbit anti-CXCR4 (1 : 150) and mouse anti-TXNIP (1 : 150), then washed for 10 min at 3 times, and fluorescence secondary antibody was added for incubation of 1 h at 25°C. After DAPI was applied for 5 min, the sections were captured using a Leica 4000 microscope (Leica, CA, Germany).

### 2.4. ROS Assay

Reactive oxygen species (ROS) were detected with OxiSelect™ In Vitro ROS/RNS Assay Kit (Cell Biolabs Inc., San Diego, CA, USA). In brief, after mice were sacrificed, the kidney tissues were promptly taken out and homogenized two times for 28 s with 30 s intervals using MM400 (Retsch GmbH, Haan, Germany) in ice-cold 1/20 (*w/v*) 50 mM PBS (pH 7.2). Homogenates were centrifuged at 10000*g* for 5 min to obtain the supernatants for measuring ROS content. 50 *μ*l (*V*_homogenate : PBS_ = 1 : 4) homogenate sample was added to the well of a 96-well plate for fluorescence assay; then 50 *μ*l of catalyst was added to each well, mixed wells, and incubated 5 minutes at room temperature. 100 *μ*l of fluorescent probe 2′,7′-dichlorofluorescin diacetate (DCFH-DA) was added to each well and incubated at room temperature under dark conditions. After 30 min of further incubation, the conversion of DCFH-DA to the fluorescent product DCF was assayed using a spectrofluorometer with excitation at 484 nm and emission at 530 nm. Blanks were included to correct for background fluorescence (conversion of DCFH-DA in the absence of homogenate). ROS formation was quantified from a DCF standard curve. Data are expressed as nmol of DCF formed per minute per mg of protein.

### 2.5. Immunoprecipitation Assay

30 mg kidney tissue was homogenized in cold protein immunoprecipitation buffer including 1x pH 7.2 PBS, 1% Triton X-100, and the protease inhibitor. The tissues were homogenized two times for 28 s with 30 s intervals using MM400 (Retsch GmbH, Haan, Germany) and centrifuged at 14000*g* for 30 min at 4°C to obtain the supernatants. After the supernatants were carried out to detect protein content, 100 *μ*g protein supernatant was added 10 *μ*l of suspended volume of protein A/G plus agarose and reared for 30 min to wipe off the nonspecific adsorption. Then the supernatants were added 5 *μ*g CXCR4 or TXNIP primary antibody and incubated for 1 h at 4°C on a rotating device. And 20 *μ*l of resuspended volume of protein A/G plus agarose was added and incubated at 4°C overnight. The immunoprecipitates were collected to centrifugate at 1000*g* for 5 min at 4°C. The pellets were washed for 4 times with 1 ml immunoprecipation buffer, each time repeating centrifugation step above. After the final wash, the pellets were resuspended in 40 *μ*l of 1 × electrophoresis sample buffer. The samples were boiled for 3 min and used to measure the level of TXNIP or CXCR4 by western blot.

### 2.6. Western Blot Analysis

Western blot analyses were performed according to a previously published method [23]. NF-*κ*B levels in the cytoplasm and nuclear extracts of kidney tissues were assayed by western blot, which were obtained by a nuclear/cytoplasm fractionation kit (BioVision Inc., USA). Protein contents of the supernatants were detected by the bicinchoninic acid assay kit (Pierece Biotechnology Inc., Rockford, IL, USA).

Western blot analyses were performed to detect primary antibodies, respectively: rabbit anti-NF-*κ*B, induced nitric oxide synthase (iNOS), and cyclooxygenase-2 (COX-2) (Cell Signaling Technology Inc., Beverly, MA); rabbit anti-CXCR4, apoptosis-associated speck-like protein containing a CARD (ASC) and cleaved caspase-1, goat anti-interleukin 1 beta (IL-1*β*), mouse anti-TXNIP, chemokine stromal-derived factor-1(SDF-1), and pro-caspase-1 (purchased from Santa Cruz Biotechnology, Inc., California, USA); rabbit anti-NLRP3 (Abcam, Cambridge, UK); and mouse anti-*β*-actin (Chemicon, California, USA).

Protein bands were measured using horseradish peroxidase- (HRP-) conjugated secondary antibodies (Cell Signaling Technology, Danvers, MA, USA) and were obtained using FluorChem MTM system (Protein Simple, CA, USA). The mean optical density (OD) values of protein bands were assessed with Scion image analysis software (Scion Corp., Frederick, MD, USA) and were normalized to mouse anti-*β*-actin or H3 as internal controls (OD detected protein/OD internal control).

### 2.7. Statistical Analysis

Data were expressed as the mean ± SEM. All the data were analyzed by the software SPSS 15.0 (SPSS Software Inc., Chicago, IL, USA) statistically. ROS level, ACR level, and western blotting results were analyzed with one-way ANOVA followed by Tukey's HSD post hoc test. *P* < 0.05 was considered to be significant.

## 3. Results

### 3.1. Troxerutin Reduces Kidney ACR, ROS, and Inflammatory Lesion in BDE-47-Treated Mice

**Figure 1:**
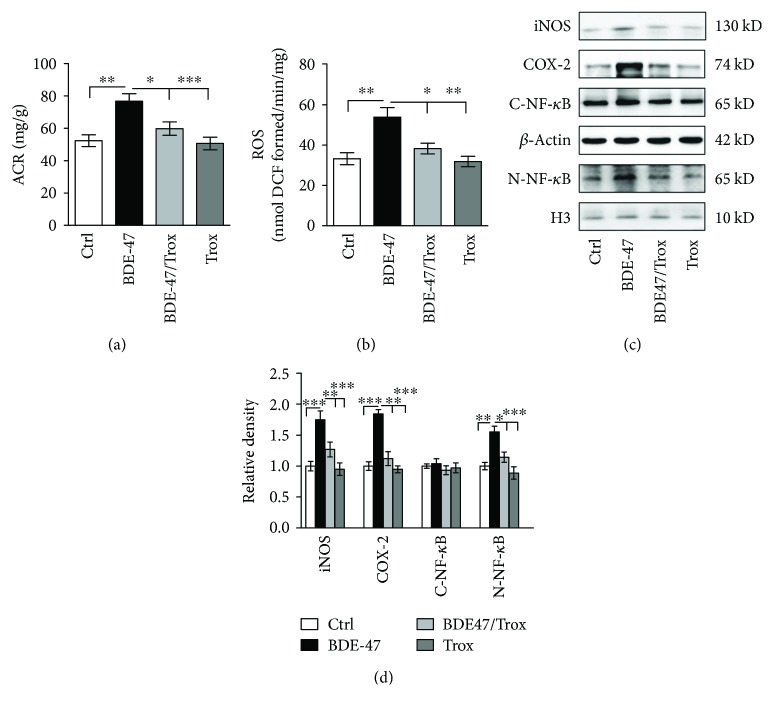
Troxerutin reduces ACR, ROS, and inflammatory lesion in the kidney of BDE-47-treated mice. (a) ACR level (albumin-to-creatinine, mg/g; *n* = 8) in BDE-47 mice including BDE-47 feeding and BDE-47/troxerutin were performed at 8 weeks. (b) ROS production was detected by fluorescent probe DCFH-DA. (c) Cytoplasm and nucleus NF-*κ*B p65, iNOS, and COX-2 were examined by western blot. (d) shows the relative density analysis of cytoplasm and nucleus NF-*κ*B p65, iNOS, and COX-2. The relative density is expressed as the ratio (iNOS/*β*-actin, COX-2/*β*-actin, cytoplasm NF-*κ*B p65/*β*-actin, and nucleus NF-*κ*B p65/H3). The control group is regarded as “1,” and all data are obtained at 8 weeks, which were the same as followed results of western blot. ^∗^ *P* < 0.05, ^∗∗^ *P* < 0.01, and ^∗∗∗^ *P* < 0.001 versus the BDE-47 group (*n* = 5).

Urine albumin-to-creatinine is suggested for albuminuria screening to assess kidney diseases and related with glomerular damage and progressive renal dysfunction [24]. Firstly, we detected the change of urinary albumin-to-creatinine ratio. The data showed that BDE-47 administration markedly elevated the ACR, whereas troxerutin inhibited urinary albumin production induced by BDE-47 (Figure 1(a)). Then to elucidate the protective mechanism of troxerutin against mouse kidney injury caused by BDE-47, we measured the activity of the NF-*κ*B pathway, which plays an important part in various cellular processes including survival, proliferation, apoptosis, linking oxidative stress, and inflammatory response [25]. As shown in Figures 1(b) and 1(c), BDE-47 treatment remarkably accelerated kidney ROS formation (Figure 1(b)) and activated the NF-*κ*B signaling pathway in the mice, promoting the translocation of NF-*κ*B from the cytoplasm into the nucleus and subsequently enhancing the activities of downstream inflammation targets COX-2 and iNOS (Figures 1(c) and 1(d)). Comfortably, troxerutin administration for 8 weeks prominently blocked these adverse changes in the kidney tissue of BDE-47-treated mice. Compared to the control group, both the troxerutin group and the BDE-47/troxerutin group had no observable diversity.

### 3.2. Troxerutin Suppresses the Activity of NLRP3 Inflammasome Caused by BDE-47

**Figure 2:**
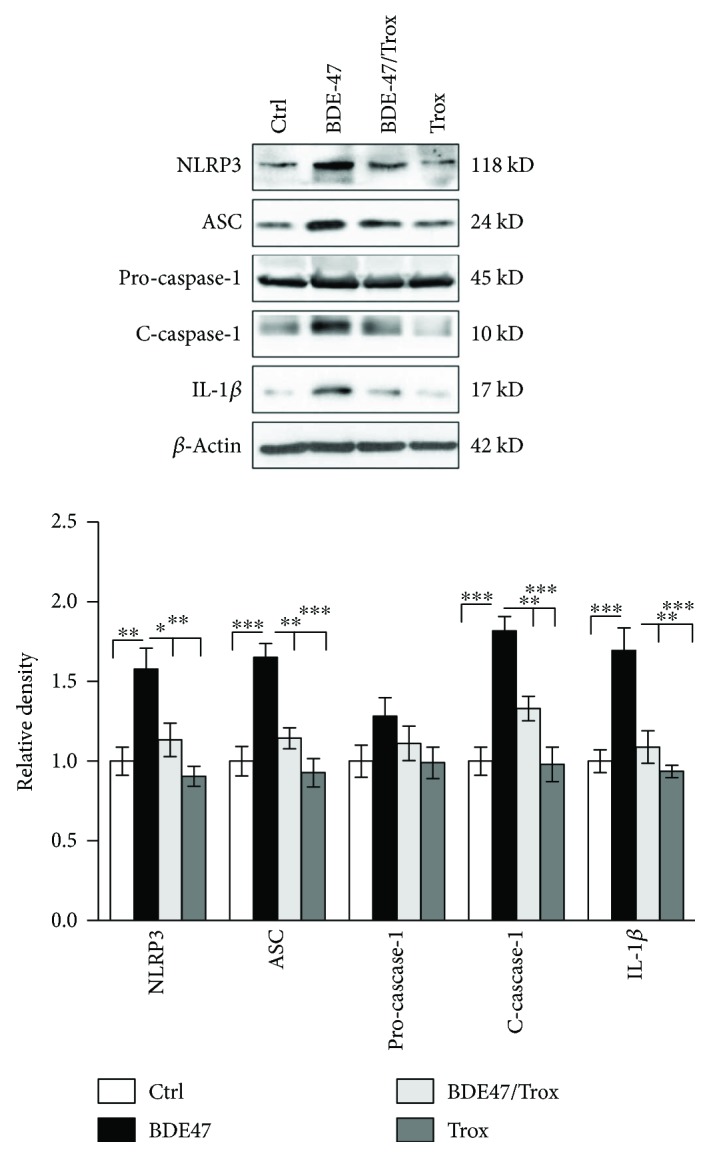
Troxerutin suppresses the activity of kidney inflammasome caused by BDE-47. NLRP3, ASC, pro-caspase-1, cleaved caspase-1 (c-caspase-1), and IL-1*β* were assessed by western blot analysis. The relative density is normalized to *β*-actin. Data are expressed as the means ± S.E.M. ^∗^ *P* < 0.05, ^∗∗^ *P* < 0.01, and ^∗∗∗^ *P* < 0.001 versus the BDE-47 group (*n* = 5).

Nucleotide-binding oligomerization domain-like receptors (NLRs) play a vital role in innate immunity involved in some chronic kidney diseases such as diabetic nephropathy [26, 27]; NLRP3 with a pyrin domain 3, as one important component of NLRs, is a key protein of inflammasome complex consisting of NLRP3, ASC, and pro-caspase-1. NLRP3 inflammasome activation results in the cleaving of pro-caspase-1 and the secretion of mature IL-1*β*, initiating inflammatory response. As shown in Figure 2, compared with the control group, BDE-47 administration markedly upregulated NLRP3 and ASC expressions, leading to the significant activation of caspase-1 and the increase of IL-1*β* secretion, and there was not a notable change in the content of pro-caspase-1. Our data indicated that BDE-47 intensified NLRP3 inflammasome-mediated IL-1*β* secretion. Additionally, troxerutin suppressed NLRP3 inflammasome activation induced by BDE-47 in the kidneys of mice, while only troxerutin treatment did not significantly affect the parameters (no significance versus the control group).

### 3.3. Troxerutin Blocks the Activity of SDF-1 and CXCR4 in the Kidney of BDE-47-Treated Mice

**Figure 3:**
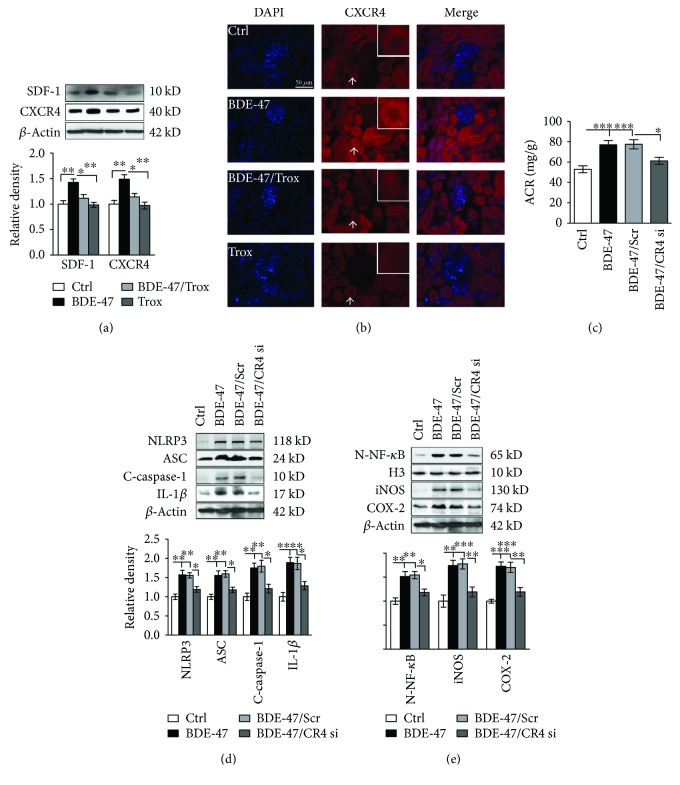
Troxerutin blocks the activity of SDF-1 and CXCR4 in the kidney of BDE-47-treated mice. (a) The expression of SDF-1 and CXCR4 was measured by western blot analysis. The relative density is normalized to *β*-actin. ^∗^ *P* < 0.05 and ^∗∗^ *P* < 0.01, respectively, means versus the BDE-47 group (*n* = 5). (b) shows renal CXCR4 immunofluorescence (Leica 4000, ×40). Nuclei were stained with DAPI (blue fluorescence). Scale bar, 50 *μ*m. White arrow indicates positive signal which was placed in the upper right corner (red). (c) CXCR4 siRNA decreased ACR in BDE-47-treated mice. ^∗^ *P* < 0.05 and ^∗∗^ *P* < 0.01 versus the adjacent groups (*n* = 8). All CR4 si means CXCR4 siRNA, and Scr means scramble. (d) CXCR4 siRNA suppressed the activity of kidney NLRP3 inflammasome in BDE-47-treated mice. ^∗^ *P* < 0.05, ^∗∗^ *P* < 0.01, and ^∗∗∗^ *P* < 0.001 versus the adjacent groups (*n* = 5). (e) CXCR4 siRNA decreased the activity of NF-*κ*B, iNOS, and COX-2. ^∗^ *P* < 0.05, ^∗∗^ *P* < 0.01, and ^∗∗∗^ *P* < 0.001 versus the adjacent groups (*n* = 5). The relative density is normalized to *β*-actin or H3 (*n* = 5).

Chemokine stromal-derived factor-1 (SDF-1 or CXCL12) and its receptor 4 (CXCR4) are critical in the generation of inflammation [28], whether SDF-1/CXCR4 mediates kidney inflammation remains unknown. We found that the treatment of BDE-47 substantially upregulated SDF-1 and CXCR4 expressions in the kidneys of mice, which were reversed after troxerutin administration (Figure 3(a)). Immunofluorescence results also showed that CXCR4 level was elevated in the cytoplasm and nucleus of glomerulus, mesangial, and duct cells of BDE-47 mice, especially in duct cells (Figure 3(b)). Furthermore, we want to know whether SDF-1/CXCR4 was involved in the activation of NLRP3 inflammasome and inflammation process induced by BDE-47. The result showed that the subcutaneous injection of CXCR4 siRNA significantly inhibited the protein expression of NLRP3 and ASC, the cleavage of pro-caspase-1, and the secretion of mature IL-1*β* in the kidney tissue of BDE-47-treated mice (Figure 3(d)). Similarly, CXCR4 siRNA treatment blocked the translocation of NF-*κ*B from the cytoplasm to the nucleus and suppressed the augmentation of iNOS and COX-2 in the kidney tissue of BDE-47-treated mice (Figure 3(e)), resulting in the decrease of kidney ACR (Figure 3(c)). These data suggested that troxerutin protected kidney injury induced by BDE-47, partly by negatively regulating the SDF/CXCR4-NLRP3 inflammasome pathway.

### 3.4. Troxerutin Inhibits the Expression of TXNIP in the Kidney of BDE-47-Treated Mice

**Figure 4:**
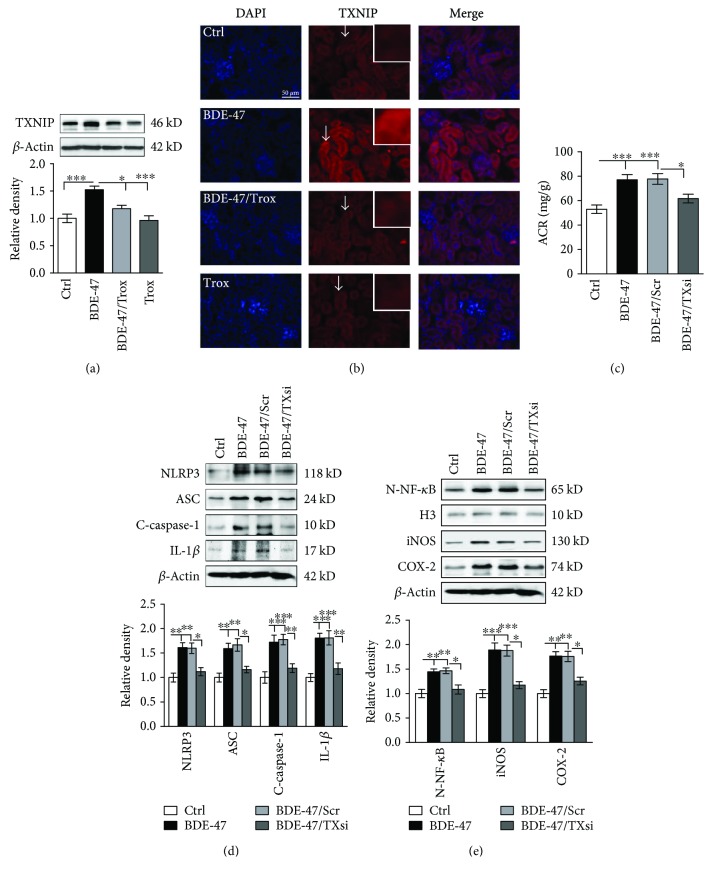
Troxerutin inhibited TXNIP and ameliorated kidney function in BDE-47 mice. (a) TXNIP expression was detected by western blot analysis. The relative density is normalized to *β*-actin. ^∗^ *P* < 0.05 and ^∗∗∗^ *P* < 0.001, respectively, means versus the BDE-47 group (*n* = 5). (b) shows renal TXNIP immunofluorescence (Leica 4000, ×40). Nuclei were stained with DAPI (blue fluorescence). Scale bar, 50 *μ*m. White arrows indicate positive signal which was placed in the upper right corner (red). (c) TXNIP siRNA decreased ACR in BDE-47-treated mice. TXsi means TXNIP siRNA, and Scr means scramble. ^∗^ *P* < 0.05 and ^∗∗∗^ *P* < 0.001 versus the adjacent groups (*n* = 8). (d) TXNIP siRNA suppressed the activity of kidney NLRP3 inflammasome in BDE-47-treated mice. ^∗^ *P* < 0.05, ^∗∗^ *P* < 0.01, and ^∗∗∗^ *P* < 0.001 versus the adjacent groups (*n* = 5). (e) TXNIP siRNA decreased the activity of NF-*κ*B, iNOS, and COX-2. ^∗^ *P* < 0.05, ^∗∗^ *P* < 0.01, and ^∗∗∗^ *P* < 0.001 versus the adjacent groups (*n* = 5).

TXNIP, a thioredoxin-binding protein, which negatively modulates thioredoxin and augments ROS accumulation, is closely associated with oxidative stress and inflammation [29]. As shown in Figure 4(a), BDE-47 caused the upregulation of kidney TXNIP protein in the mice. Immunofluorescence results also showed TXNIP was significantly increased in glomerulus, mesangial, and duct cells of BDE-47 mice (Figure 4(b)). Importantly, troxerutin inhibited the alteration; meanwhile, downregulation of TXNIP with siRNA also could inhibit ACR production (Figure 4(c)) and reduce the activation of NLRP3 inflammasome and the secretion of IL-1*β* (Figure 4(d)), resulting in the low expression of iNOS and COX-2 (Figure 4(e)). These data revealed that troxerutin may decrease kidney lesions induced by BDE-47 via suppressing TXNIP-regulated NLRP3 inflammasome activation.

### 3.5. Troxerutin Ameliorates Kidney Inflammation by CXCR4 Targeting TXNIP in BDE-47-Treated Mice

**Figure 5:**
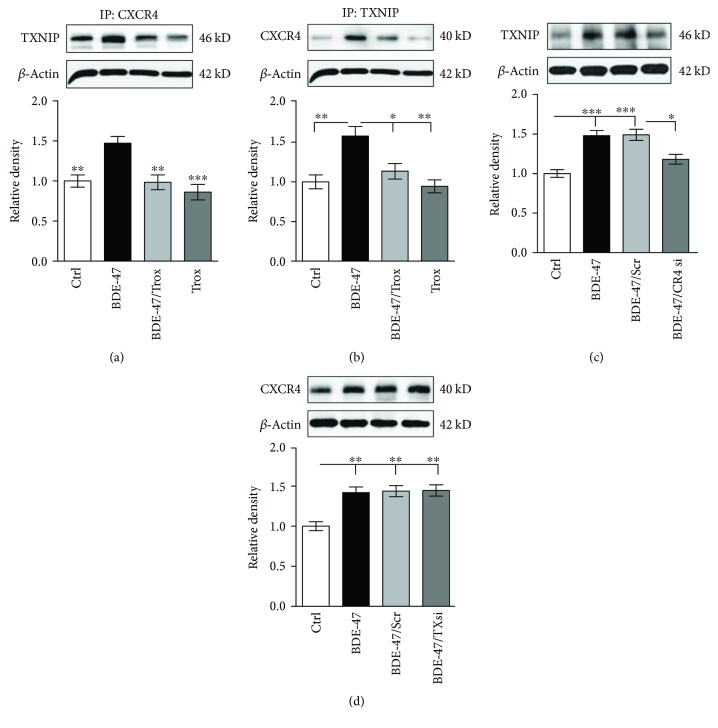
Troxerutin inhibited the interaction of CXCR4 and TXNIP in the kidney of BDE-47 mice. (a) TXNIP level was detected by western blot analysis after CXCR4 was immunoprecipitated with anti-CXCR4. ^∗∗^ *P* < 0.01 and ^∗∗∗^ *P* < 0.001 versus the BDE-47 group (*n* = 5). (b) CXCR4 level was measured by western blot analysis after TXNIP immunoprecipitation. ^∗^ *P* < 0.05 and ^∗∗^ *P* < 0.01 versus the BDE-47 group (*n* = 5). (c) CXCR4 siRNA decreased the expression of TXNIP in BDE-47-treated mice. ^∗^ *P* < 0.05 and ^∗∗∗^ *P* < 0.001 versus the adjacent groups. (*n* = 5). (d) TXNIP siRNA did not change the level of CXCR4 in BDE-47-treated mice. ^∗∗^ *P* < 0.01 versus the BDE-47/Scr group (*n* = 5). CR4 si: CXCR4 siRNA; TXsi: TXNIP siRNA; Scr: scramble.

The fact that both CXCR4 and TXNIP activated NLRP3 inflammasome and participated in inflammatory reactions promotes us to investigate whether there is a direct connection between CXCR4 and TXNIP via protein coimmunoprecipitation. The results revealed CXCR4 antibody could pull down TXNIP protein (Figure 5(a)). Similarly, TXNIP antibody could drag CXCR4 protein (Figure 5(b)). Furthermore, CXCR4 siRNA decreased the expression level of TXNIP protein (Figure 5(c)), while TXNIP siRNA did not affect CXCR4 expression in the kidney of BDE-47 mice (Figure 5(d)). The data suggested the CXCR4-TXNIP interaction was involved in the process of kidney impairment induced by BDE-47.

## 4. Discussion

The persistent environmental pollutants PBDEs have been demonstrated to have various disadvantage effects such as the induction of toxicity in the brain, liver, renal, and endocrine systems. Substantial research suggested that the PBDE toxicity was strongly associated with PBDE-induced oxidative stress and inflammatory reactions. Interestingly, dietary sources of beneficial antioxidants such as flavonoids, *α*-tocopherol, and omega-3 polyunsaturated fatty acids could protect tissue cells against the toxicity of PBDEs, involved in mitochondrial electron transport system dysfunction, DNA fragmentation, and endoplasmic reticulum stress via blocking the generation of ROS [7, 30, 31]. Troxerutin, a natural flavonoid, possesses the efficacy of antioxidation and anti-inflammation. In this study, we demonstrated that troxerutin worked well in the protection of kidney injury induced by BDE-47 via attenuating oxidative stress-mediated NLRP3 inflammasome activation.

Inflammasome is involved in the progression of inflammation through regulating the expression of proinflammatory factors. NLRP3 inflammasome, including NLRP3 with a pyrin domain 3, ASC, and pro-caspase-1, provokes profound inflammatory reactions and contributes to chronic nephropathies and other health disorders [27, 32]. Consequently, NLRP3 inflammasome has been regarded as a danger signal sensor which correlates oxidative stress and inflammatory reaction. There is a strong relationship between NLRP3 inflammasome and TXNIP in the development of kidney diseases [33]. Inflammasome activators can induce ROS production, which arouses the dissociation of TXNIP from thioredoxin; then TXNIP binds and activates NLRP3 inflammasome, which triggers the cleavage of pro-caspase-1 into active caspase-1 and, subsequently, the secretion of mature IL-1*β*. IL-1*β* has the ability to induce consecutive inflammatory response, such as activating NF-*κ*B and downstream molecules iNOS and COX-2 [3]. Increasing knowledge shows that BDE-47 elevates ROS generation via depletion of the expressions of antioxidant response genes and the content of glutathione (GSH) [30, 34]. Excessive ROS leads to the occurrence and development of inflammatory response [7]. As a result, BDE-47 augments the nuclear translocation of NF-*κ*B p65 in the liver, promotes the release of IL-6 and IL-8 [10], and increases the activity of COX-2 in human extravillous trophoblast cell line [31]. Our results also showed that BDE-47 induced ROS accumulation and TXNIP expression and then activated NLRP3 inflammasome and promoted mature IL-1*β* secretion; in turn, triggered the translocation of NF-*κ*B p65 from the cytoplasm into the nuclear of kidney cells in the mice, advanced the expression of downstream inflammatory factors such as iNOS and COX-2, and ultimately enhanced urine protein production. Interestingly, both troxerutin and TXNIP siRNA could decrease these adverse parameters, ameliorated kidney function, which is associated with troxerutin inhibiting the activity of NLRP3 inflammasome induced by D-glucose in HUVECs [21].

ROS accumulation also stimulates the expression of SDF-1 and its receptor CXCR4 [35]. SDF-1/CXCR4 axis can induce intracellular signalings involved in inflammatory response, cell survival, and proliferation, playing a vital role in some diseases such as kidney diseases and systemic lupus erythematosus [36, 37]. CXCR4, as a surrogate marker for autoimmunity, has been implicated in kidney homeostasis and regeneration and is significantly increased in kidney tumor and human kidney biopsies from patients with proteinuria kidney diseases [36, 38]. Here, we found that BDE-47 strongly elevated the protein expressions of SDF-1 and CXCR4 in the kidney cells of mice, whereas troxerutin weakened the abnormal alteration induced by BDE-47. Furthermore, AMD3100, an antagonist of CXCR4, has been approved to ameliorate kidney function and exhibits a protective effect on acute kidney injury and chronic kidney disease [39–41]; we also found that CXCR4 siRNA and AMD3100 effectively downregulated the NLRP3 pathway, decreased the expression of inflammatory cytokines iNOS and COX-2, and improved kidney dysfunction induced by BDE-47. Interestingly, a previous report reveals that there is a tight contact between transcript factors NF-*κ*B and CXCR4 expression in nonalcoholic steatohepatitis [42]. We also found that CXCR4 siRNA obviously inhibited NF-*κ*B activity. Our results demonstrated that troxerutin can regulate NLRP3 activity via targeting SDF-1/CXCR4 in the kidney of BDE-47-treated mice. Additionally, we utilized coimmunoprecipitation method to verify the direct connection between CXCR4 and TXNIP. Moreover, CXCR4 siRNA reduced the expression of TXNIP in the kidney cells of BDE-47-treated mice; however, TXNIP siRNA did not significantly alter the expression of CXCR4. In immunofluorescence stain, we tried for several times to colabel TXNIP and CXCR4, and there were no results which may be due to steric hindrance. Consequently, our findings revealed that troxerutin ameliorated kidney dysfunction caused by BDE-47 via regulating CXCR4-TXNIP/NLRP3 inflammasome.

In summary, the present study firstly provides the data that CXCR4/TXNIP is involved in the modulation of the kidney damage via directly regulating NLRP3 inflammasome. Based on our results, NLRP3 may represent a potential target for the prevention and treatment of kidney lesion induced by environmental toxins BDE-47 via ROS/CXCR4-TXNIP-dependent modulation style. Importantly, troxerutin can availably block the aberrant parameters and ameliorate kidney function through the above-mentioned signaling pathway. However, in future research, it still needs to determine how CXCR4 mediates the expression of TXNIP, as it contributes well to the understanding of the fundamental mechanism underlying the toxicity of environmental toxins BDE-47 and the protective efficacy of troxerutin.

## Abbreviations

ROS: Reactive oxygen species
DCFH-DA: 2′,7′-Dichlorofluorescin diacetate
BDE-47: Tetra-brominated diphenyl ethers
PBDEs: Polybrominated diphenyl ethers
NLRs: Nucleotide-binding oligomerization domain-like receptors
NLRP3: Nucleotide-binding oligomerization domain-like receptors (NLR) protein 3 containing pyrin domain
ASC: Apoptosis-associated speck-like protein containing a CARD
IL-1*β*: Interleukin 1 beta
AMD3100: 1′-[1,4-Phenylenebis (methylene)] bis-1,4,8,11-tetraazacyclotetradecane octahydrochloride
PBS: Phosphate-buffered saline
BSA: Bovine serum albumin
NF-*κ*B: Nuclear factor kappa B
iNOS: Nitric oxide synthase
COX-2: Cyclooxygenase-2
ACR: Albumin-to-creatinine ratio
SDF-1: Chemokine stromal-derived factor-1
CXCL12: C-X-C chemokine ligand 12
CXCR4: CXCL12 receptor 4
TXNIP: Thioredoxin-interacting protein
HUVECs: Human umbilical vein endothelial cells
GSH: Glutathione.

## Supplementary Materials

Supplementary Materials Figure S1: different doses of BDE-47 increased ACR production and kidney ROS accumulation in the mice. (A) The results of ACR (urine albumin-to-creatinine) after BDE-47 were given by gavage for 8 weeks (*n* = 8). (B) Kidney ROS content was detected by fluorescent probe DCFH-DA after BDE-47 was administrated by gavage for 8 weeks (*n* = 5). ^∗^ *P* < 0.05 and ^∗∗∗^ *P* < 0.001 versus the control (Ctrl) group. The data were analyzed with one-way ANOVA followed by the post hoc Tukey test. Figure S2: different doses of troxerutin reduced ACR production and kidney ROS content induced by BDE-47 in the mice. (A) The results of ACR after BDE-47 (50 mg/kg/day) and troxerutin were administrated by gavage for 8 weeks (*n* = 8). (B) Kidney ROS level was detected DCFH-DA after BDE-47 and troxerutin were orally given for 8 weeks (*n* = 5). ^∗^ *P* < 0.05, ^∗^ *P* < 0.01, and ^∗∗∗^ *P* < 0.001 versus the BDE-47 group. The data were analyzed with one-way ANOVA followed by the post hoc Tukey test. Figure S3: different doses of AMD3100 reduced ACR production and kidney CXCR4 expression induced by BDE-47 in the mice. (A) The results of ACR after BDE-47 (50 mg/kg/day) were administrated for 8 weeks, and AMD3100 was subcutaneously given for 4 weeks (*n* = 8). (B) Kidney CXCR4 level was detected by western blot after BDE-47 was orally given for 8 weeks, and AMD3100 was subcutaneously given for 4 weeks (*n* = 5). ^∗^ *P* < 0.05, ^∗^ *P* < 0.01, and ^∗∗∗^ *P* < 0.001 versus the BDE-47 group. The data were analyzed with one-way ANOVA followed by the post hoc Tukey test.
