# Supplementary material for: Troxerutin Protects Kidney Tissue against BDE-47-Induced Inflammatory Damage through CXCR4-TXNIP/NLRP3 Signaling
Source: Oxid Med Cell Longev. 2018 Apr 19;2018:9865495. doi: 10.1155/2018/9865495 (PMC5932985; doi:10.1155/2018/9865495)
Supplement: Supplementary Materials — Figure S1: different doses of BDE-47 increased ACR production and kidney ROS accumulation in the mice. (A) The results of ACR (urine albumin-to-creatinine) after BDE-47 were given by gavage for 8 weeks (n = 8). (B) Kidney ROS content was detected by fluorescent probe DCFH-DA after BDE-47 was administrated by gavage for 8 weeks (n = 5). ∗ P < 0.05 and ∗∗∗ P < 0.001 versus the control (Ctrl) group. The data were analyzed with one-way ANOVA followed by the post hoc Tukey test. Figure S2: different doses of troxerutin reduced ACR production and kidney ROS content induced by BDE-47 in the mice. (A) The results of ACR after BDE-47 (50 mg/kg/day) and troxerutin were administrated by gavage for 8 weeks (n = 8). (B) Kidney ROS level was detected DCFH-DA after BDE-47 and troxerutin were orally given for 8 weeks (n = 5). ∗ P < 0.05, ∗ P < 0.01, and ∗∗∗ P < 0.001 versus the BDE-47 group. The data were analyzed with one-way ANOVA followed by the post hoc Tukey test. Figure S3: different doses of AMD3100 reduced ACR production and kidney CXCR4 expression induced by BDE-47 in the mice. (A) The results of ACR after BDE-47 (50 mg/kg/day) were administrated for 8 weeks, and AMD3100 was subcutaneously given for 4 weeks (n = 8). (B) Kidney CXCR4 level was detected by western blot after BDE-47 was orally given for 8 weeks, and AMD3100 was subcutaneously given for 4 weeks (n = 5). ∗ P < 0.05, ∗ P < 0.01, and ∗∗∗ P < 0.001 versus the BDE-47 group. The data were analyzed with one-way ANOVA followed by the post hoc Tukey test. [file 9865495.f1.docx]

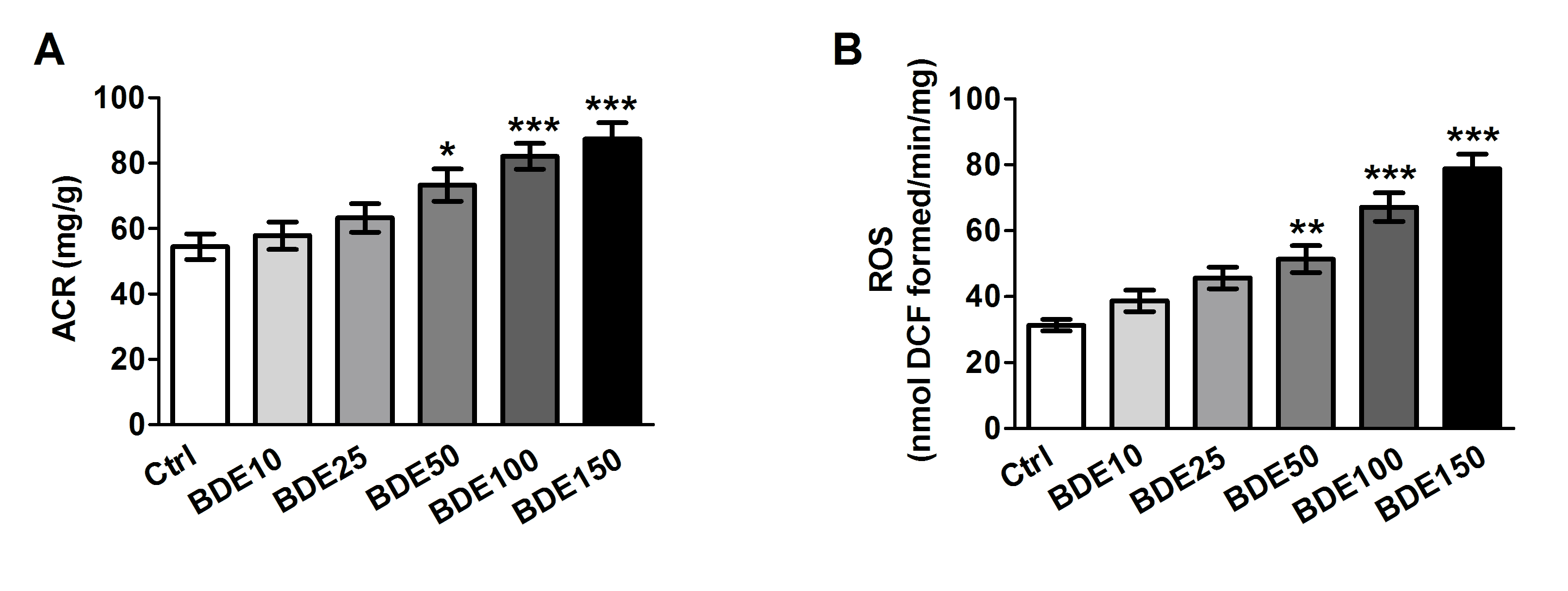


**Figure S1.** Different doses of BDE-47 increased ACR production and kidney ROS accumulation in the mice. (A) The results of ACR ratio (Urine albumin-to-creatinine) after BDE-47 were given by gavage for 8 weeks (n=8). (B) Kidney ROS content was detected by fluorescent probe DCFH-DA after BDE-47 were administrated by gavage for 8 weeks (n = 5). *P < 0.05 and ***P < 0.001 versus control (Ctrl) group. The data were analyzed with One-way ANOVA followed by post hoc Tukey test.


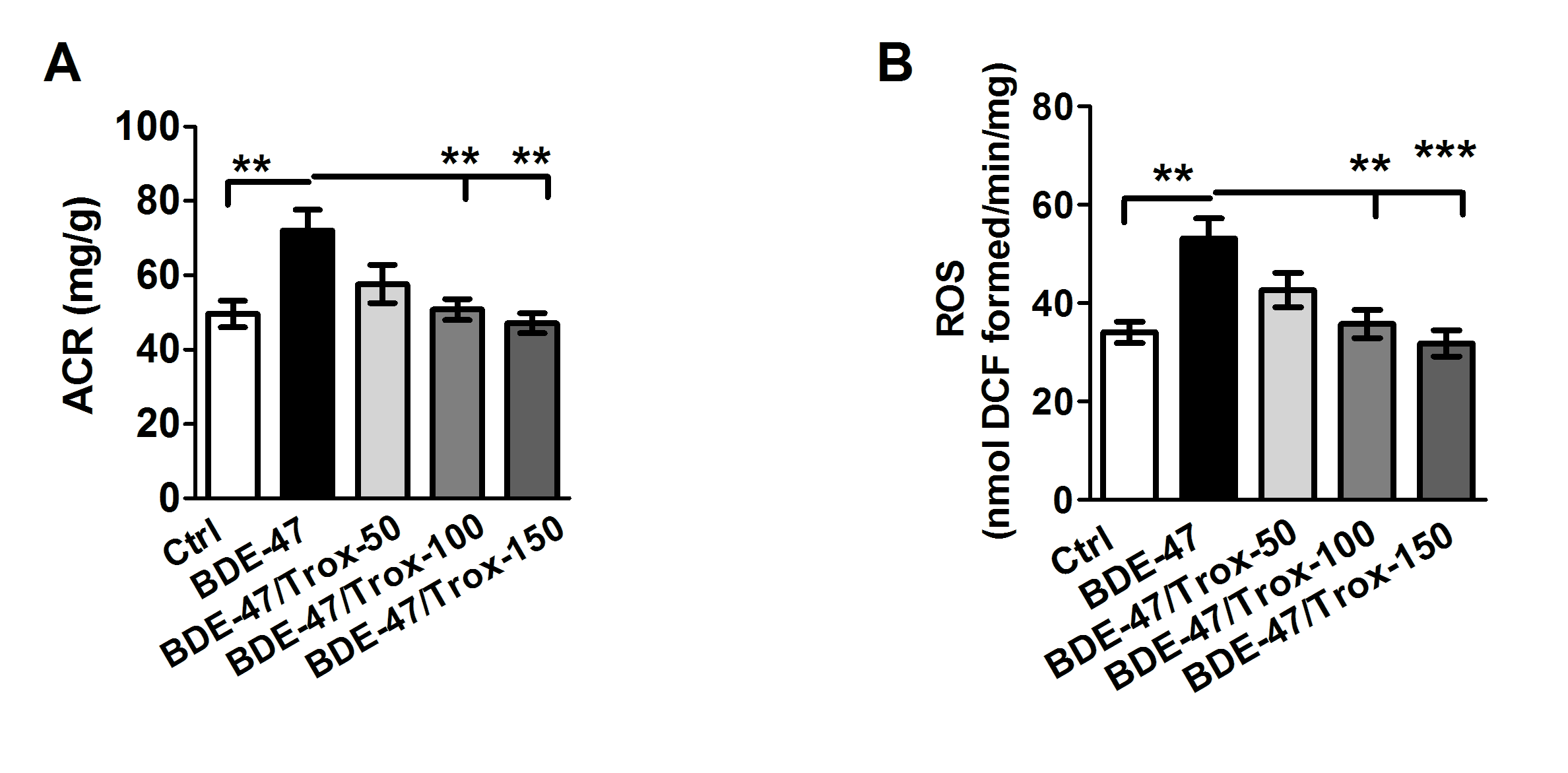


Figure S2. Different doses of Troxerutin reduced ACR production and kidney ROS content induced by BDE-47 in the mice. (A) The results of ACR ratio after BDE-47 (50mg/kg/day) and Troxerutin were administrated by gavage for 8 weeks (n=8). (B) Kidney ROS level was detected DCFH-DA after BDE-47 and Troxerutin were orally given for 8 weeks (n= 5). *P < 0.05, *P<0.01 and ***P < 0.001 versus BDE-47 group. The data were analyzed with One-way ANOVA followed by post hoc Tukey test.


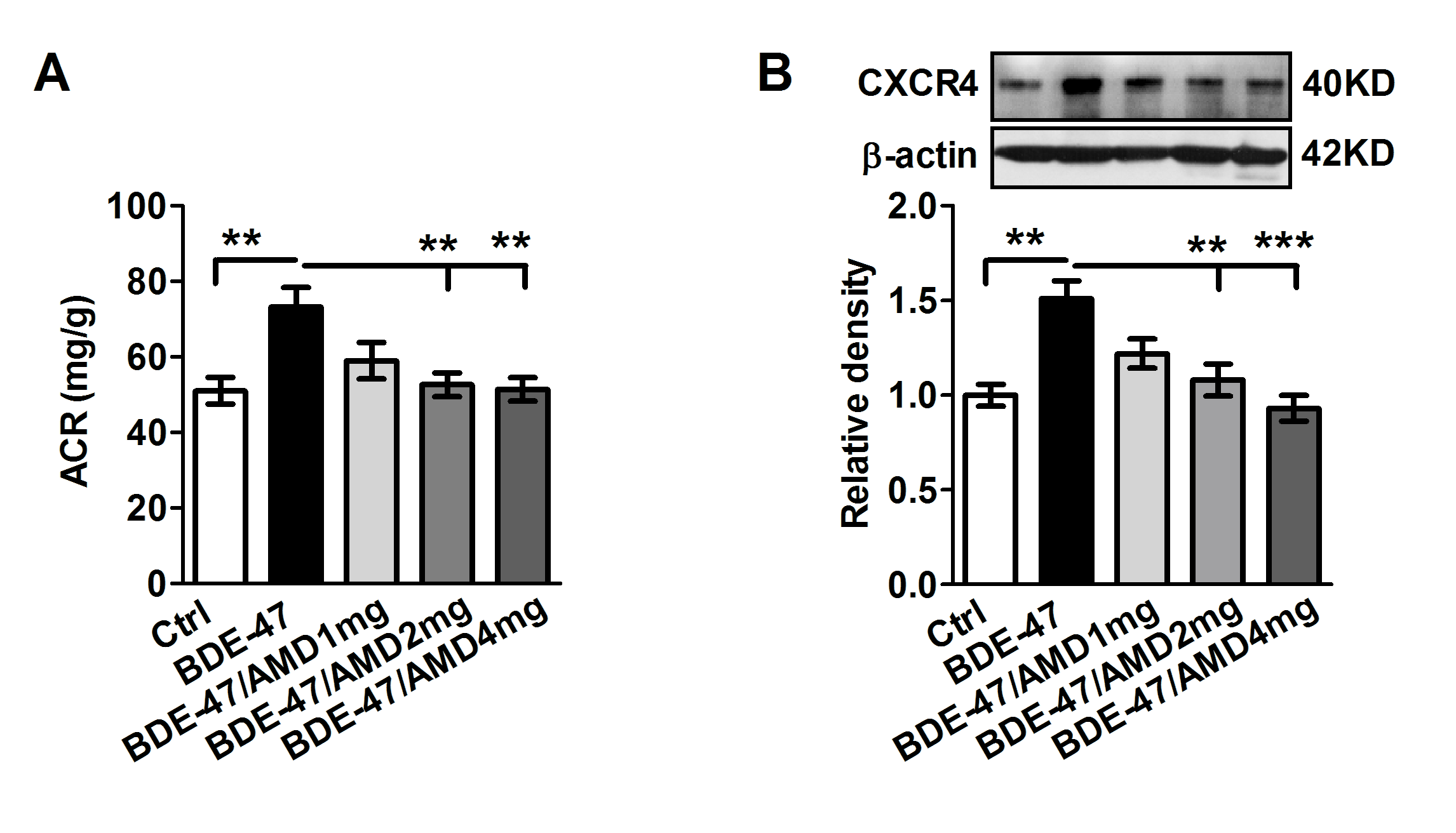


Figure S3. Different doses of AMD3100 reduced ACR production and kidney CXCR4 expression induced by BDE-47 in the mice. (A) The results of ACR ratio after BDE-47 (50mg/kg/day) was administrated for 8 weeks and AMD3100 were subcutaneously given for 4 weeks (n=8). (B) Kidney CXCR4 level was detected by western blot after BDE-47 was orally given for 8 weeks and AMD3100 were subcutaneously given for 4 weeks (n= 5). *P < 0.05, *P<0.01 and ***P < 0.001 versus BDE-47 group. The data were analyzed with One-way ANOVA followed by post hoc Tukey test.
